# Deciphering the Basis of Molecular Biology of Selected Cardiovascular Diseases: A View on Network Medicine

**DOI:** 10.3390/ijms231911421

**Published:** 2022-09-28

**Authors:** Noora Alhajri, Mohammad Rustom, Adedayo Adegbile, Weshah Ahmed, Salsabeel Kilidar, Nariman Afify

**Affiliations:** 1 Department of Internal Medicine, Cleveland Clinic Abu Dhabi (CCAD), Abu Dhabi P.O. Box 112412, United Arab Emirates; 2 College of Medicine and Health Sciences, Khalifa University, Abu Dhabi P.O. Box 127788, United Arab Emirates; 3 Department of Emergency Medicine, Sheikh Shakhbout Medical City SSMC, Abu Dhabi P.O. Box 11001, United Arab Emirates

**Keywords:** network medicine, cardiovascular disease (CVD), molecular biology, gene therapy, system biology

## Abstract

Cardiovascular diseases are the leading cause of death across the world. For decades, researchers have been studying the causes of cardiovascular disease, yet many of them remain undiscovered or poorly understood. Network medicine is a recently expanding, integrative field that attempts to elucidate this issue by conceiving of disease as the result of disruptive links between multiple interconnected biological components. Still in its nascent stages, this revolutionary application of network science facilitated a number of important discoveries in complex disease mechanisms. As methodologies become more advanced, network medicine harbors the potential to expound on the molecular and genetic complexities of disease to differentiate how these intricacies govern disease manifestations, prognosis, and therapy. This is of paramount importance for confronting the incredible challenges of current and future cardiovascular disease research. In this review, we summarize the principal molecular and genetic mechanisms of common cardiac pathophysiologies as well as discuss the existing knowledge on therapeutic strategies to prevent, halt, or reverse these pathologies.

## 1. Introduction

The modern application of scientific knowledge to understand the mechanism of disease pathophysiology for the delivery of efficient therapeutic interventions has been guided by the “Oslerian Principles of Disease Definition” (Osler, William. The principles and practice of medicine. D. Appleton and Company, 1912). The “Oslerian Principle” defines the disease process by clinical and pathological correlations, and now in the modern era, by the molecular or genetic alterations that contribute to the disease presentation. Inherent to this principle are fundamental concepts that specific key factors function in simple molecular circuits that regulate and control disease pathology, including where interventions can target these molecules to ameliorate disease processes [1]. 

Although the Oslerian principle has increased our appreciation and understanding of disease processes at the molecular level, it oversimplifies the underlying basis of certain pathologies and thus limits our full understanding of the complex interaction of multiple factors that contribute to the disease process. Since the advent of molecular biology, it has become increasingly clear that a single gene mutation can result in disease pathogenesis. However, disease manifestations rarely result from an individualized abnormality in an effector; rather they are almost always the net result of interaction between multiple pathways that interact through a complex network composed of numerous molecules and genes [2,3].

Molecular cardiology is an innovative and rapidly expanding branch of molecular biology that aims to use results from translational cardiovascular research to diagnose, treat, and prevent cardiovascular diseases. This emerging discipline has increased our knowledge and understanding of the molecular mechanisms of various cardiovascular diseases and provided novel diagnostic and therapeutic tools. For instance, molecular and genetic cardiology has increased the awareness of possible mechanisms of inheritance of certain chronic cardiac diseases (e.g., heart failure and congenital heart anomalies) and their links to specific gene mutations [4,5,6]. In this modern era of molecular medicine, we now have a framework of potential interaction between complex pathways and molecules known as the “interactome”. The interactome describes the network of interactions between various molecules in the human cells. It consists of nearly 1000 metabolites, 25,000 protein-coding genes, and a significant number of functional RNA molecules, post-translationally modified proteins, and other cellular components, together exceeding 100,000 molecules [7].

A network-based approach in cardiovascular disease could have several implications for the management and diagnosis of these diseases. A better understanding of the cellular interconnectedness of disease pathogenesis and progression could ease the identification of genes and cellular pathways, in return offering better options for tailored therapeutic interventions. It is widely recognized now that the application of network theory can aid our understanding of disease manifestation and possibly, when manipulated, control disease phenotype and prognosis. These approaches that systematically explore the net interaction between complex molecules at a cellular level and their relationship to distinct phenotypes of a disease are frequently recognized as “network medicine” [1]. These advances could also revolutionize clinical practice through a better identification of crucial biomarkers in disease pathogenesis, leading to a more accurate disease classification and management, paving the way for personalized medicine. 

We are in the midst of a global cardiovascular disease epidemic. According to the GBD study, the prevalence of total cardiovascular diseases (CVDs) doubled from 271 million in 1990 to 523 million in 2019, and the total number of CVD deaths increased from 12.1 million in 1990 to 18.6 million in 2019 [8]. Cardiovascular diseases continue to be the leading cause of disease burden worldwide. Our aim here is to provide an overarching review of the fundamental cellular networks involved in cardiovascular disease. These principles, tools, and methodologies derived from network medicine are facilitating the emergence of a new body of knowledge that paves the way for an innovative and personalized approach in disease management known as precision medicine.

### 1.1. Molecular Basis of Cardiac Development

Cardiogenesis in vivo refers to the development of the heart from mesodermal progenitor cells that form the cardiac crescent. The cardiac crescent then folds on itself to form the linear heart tube. The progenitor cells that form the linear tube are referred to as the primary heart field. These cells form the left ventricle as well as the atrial chambers [9]. A population of splanchnic mesoderm cells then latch onto the dorsal aspect of the linear heart tube at the onset of looping and are referred to as the secondary heart field. The secondary heart field serves to form the right ventricle and the outflow tract. Heart field specification is controlled by two main signaling pathways, the canonical and the noncanonical WNT/β-catenin pathways [10]. An interplay of the temporal signaling of these pathways guides progenitor cells into either a primary or a secondary heart field [11]. Steimle et al. showed that the transcription factors Nkx2.5 and Tbx5 early on in cardiogenesis are expressed in the primary heart field as a result of the activation of the canonical WNT pathway. Later throughout development, the activation of the noncanonical pathway represses the canonical pathway and leads to the expression of secondary heart field transcription factors such as Islet 1 and Hand 2 [12]. The size of the heart fields is regulated through feedback mechanisms between the heart field transcription factors in the WNT signaling pathway, β-catenin, and retinoic acid. Kim et al. found that in retinoic acid-treated mice, the cellular proliferation of the outflow tract and the left atria was significantly reduced [13]. These studies suggest an important role of RA signaling in restricting the cardiac progenitor cells in the anterior lateral plate mesoderm. During fetal development, cardiomyocytes, the contractile cells of the heart, mature to become differentiated muscular units called sarcomeres that adhere together through protein-rich gap junctions that allow for the synchronous firing of the contractile units [14]. Cardiomyocytes exist in a complex milieu of endothelial cells, vascular smooth muscle cells, fibroblasts, and immune cells and are regulated heavily through complex biochemical and molecular pathways that govern the contractile ability and excitability of each individual unit [15]. For the heart to function properly, it requires the coordinated and synchronous effort of each individual cardiomyocyte with regards to electrical excitability and mechanical contractility. Any changes that might affect the delicate interplay of the cardiomyocytes and its surrounding environment or the cardiomyocytes within themselves can precipitate heart disease. 

### 1.2. Cardiac Metabolism in Health 

The heart is a metabolically demanding organ that requires an enormous amount of energy to perfuse the organs to sustain life. In order to maintain normal cardiac requirements, a highly regulated coupling of ATP production and cardiac contraction is essential. The heart utilizes a variety of substrates for ATP production in the mitochondrion including carbohydrates, fatty acids, amino acids, lactic acid, and ketone bodies. The major energy sources for the normal heart transitions between carbohydrates in the fed state and fatty acids in the fasting state. While carbohydrates and fatty acids are the main substrates for energy production, the cardiac network is adept at using other substrates when there is an ample source. For example, in periods of sustained fasting, blood ketone levels are increased, resulting in an increase in utilization by the heart [16].

### 1.3. Cardiac Metabolism in Exercise

Physical exercise requires greater contractility to meet the demand of oxygenated blood. In order to keep up with the demand, there needs to be continuous ATP production and increased mitochondrial calcium concentration. During exercise, lipolysis from adipose tissue and glycolysis in skeletal muscle increase the levels of fatty acids and lactate, respectively. These energy sources are then used by the heart to fuel the metabolic energy demands [17,18]. Increases in triacylglycerol are also evident in exercise and seem to be linked to the presence of lactate [19]. In addition, genes that regulate fatty acid transport and catabolism are elevated, which may be helpful in maximizing fat utilization in the heart [20,21]. The ß-adrenergic signaling pathway allows for the heart to quickly contract in order to meet the increased metabolic demands needed for exercise. When a ß-adrenergic agonist interacts with the ß receptor, G-coupled protein receptor-led changes activate adenylate cyclase to form the second messenger cyclic AMP (cAMP). Then, cAMP is phosphorylated via protein kinase A to stimulate the metabolism and also to phosphorylate the cardiac L-type calcium channel protein, increasing the likelihood that the channel will open. Calcium ions pass through the sarcolemmal channel in order to release more calcium ions from the sarcoplasmic reticulum. The calcium ions in the cytoplasm increase the rate of ATP breakdown. A rise in myosin ATPase will increase the rate of contraction, while the increased activation of troponin-C increases the peak force development.

### 1.4. Cardiac Metabolism in Disease

#### 1.4.1. Obesity

Alterations in cardiac metabolism and energy requirements are major factors in obesity-related cardiac dysfunction. While it is suggested that obesity can potentially contribute to cardiac dysfunction, the exact mechanisms behind it have yet to be determined. Recent studies offer possible changes in altered cardiac metabolism. For example, Duncan et al. described overexpression of the genes that regulate lipoprotein lipase and fatty acid transporters in patients with obesity [22]. Elevated circulating triglycerides and increased production of VLDL-triglycerides shift the body’s reliance on glucose to fatty acid metabolism [23]. This can cause a buildup of cardiotoxic fat metabolites that enter the myocardium [24,25]. The accumulation of the cardiotoxic metabolites (i.e., diacylglycerol, ceramide) can cause mitochondrial dysfunction and the generation of reactive oxygen species; this leads directly to apoptosis or indirectly through an inflammatory reaction, which can compromise the myocardium leading to a decrease in cardiac efficiency [26,27,28]). Furthermore, a proposal put forward by Young et al. suggests the downregulation of the PPARα/a decrease in PPARα responsiveness in obesity causes an underexpression of fatty acid oxidative enzymes, intracellular lipid development, and cardiomyopathy [29].

#### 1.4.2. Diabetes

Hyperglycemia can cause damage through the increased production of advanced glycosylation end products (AGEs), which are capable of deactivating nitric oxide and weakening coronary vasodilation [30]. This leads to oxidative damage to the cardiac myocytes because they lack normal amounts of free radical scavengers. In addition, oxidative stress can alter normal gene function, signal transduction, and activate cell death pathways [31]. Increased activity of the diacylglycerol-protein kinase C (DAG-PKC) pathway was identified in diabetic animals as a causative mechanism of diabetic cardiomyopathy by Way [32]. IGF-1 signaling has been implicated as a potential factor in the progression of diabetic cardiomyopathy. IGF-1 promotes skeletal muscle hypertrophy as well as a switch to glycolysis by activating the calcium calmodulin-dependent phosphatase calcineurin and inducing NFATC-1 nuclear translocation [33,34]. Qin et al. demonstrated the increased generation of arrhythmias in diabetic cardiomyopathy through RNase protection assay and Western blot analysis; there is a reduction in mRNA expression and protein density of the key cardiac potassium channel genes Kv2.1, Kv4.2, and Kv4.3 in left ventricular myocytes within 14 days of inducing type I diabetes in rats [35]. The activation of stretch receptors in the heart activates the renin-angiotensin-aldosterone system (RAAS), which can lead to cardiac remodeling, resulting in decreased cardiac performance [30]. In diabetic patients, there is an upregulation of RAAS, which increases the levels of angiotensin II that produces reactive oxygen species, which lead to oxidative damage [36]. MicroRNAs may also be implicated in diabetic cardiomyopathy. Studies have shown that miR-143, miR-181, miR-103, miR-107, and miR-802 are involved in the control of glucose metabolism and insulin sensitivity [37,38,39].

## 2. Molecular Biology of Cardiovascular Diseases

### 2.1. Cardiomyopathies

Cardiomyopathies (CMP) are a group of myocardial disorders that structurally and functionally affect the heart muscle in the absence of a disease that could better explain the extent of myocardial damage in the affected heart [40]. This definition has undergone significant changes from the time it was introduced in 1957 as we became more knowledgeable about the etiology and pathophysiology of this disease [41]. The most recent recognized classification of this disorder is the 2013 MOGE(S) nosology system was proposed by the World Heart Federation, which describes the disease not only according to the morpho-functional phenotype (M) but also according to the organ involvement (O), genetic inheritance pattern (G), etiological annotation (E) including underlying disease substrate, and functional status of the disease (S). This system offers a more flexible approach to describing the overlapping phenotypic and genetic syndromes [42].

Cardiomyopathies can have either an acquired or congenital cause and can manifest in similar ways [43]. The clinical manifestations of this disease typically involve an impairment that affects function, structure, ventricular filling, and the ejection of blood [41]. Often enough, these manifestations are severe enough to result in heart failure [43]. Different classifications of cardiomyopathies have emerged and evolved as we have gained more knowledge about the obscure etiologies and pathophysiologies of the different types of cardiomyopathies. The two most contemporary classifications are those put forth by the World Heart Federation and the American Heart Association, which classified cardiomyopathies based on morphological phenotypes and molecular genotypes and on predominant organ involvement, respectively [41]. However, for the means of this article, we will follow the classification of cardiomyopathies by the WHO. WHO recognizes six classes of cardiomyopathies. The most prevalent forms are listed below:

- Restrictive.
- Dilated.
- Hypertrophic.

### 2.2. Restrictive Cardiomyopathy

Restrictive cardiomyopathy (RCM) refers to a group of myocardial disorders characterized by diastolic dysfunction causing restrictive ventricular filling, reduced diastolic volume, and diastolic pressure overload in the presence of a preserved systolic function and myocardial thickness and in the in the absence of ischemic heart disease, hypertension, valvular disease, or congenital heart disease [43,44]. In RCM, the stiffening of the myocardium causes a disproportionately high rise in chamber pressure in response to relatively small changes in volume, causing a restrictive physiology [45]. RCM can be either primary (i.e., idiopathic RCM) or secondary to a systemic disorder. Secondary causes of RCM are further divided into infiltrative (e.g., amyloidosis, sarcoidosis) or storage disease related (e.g., hemochromatosis, Anderson Fabry’s disease) [43]. RCM may present differently in children and adults, but the most common initial manifestation of the disease is heart failure. Additional manifestations can include failure to thrive, exertional syncope, pericardial disease, stroke, atrial fibrillation, and sudden cardiac death [44].

#### Molecular and Genetic Basis of Restrictive Cardiomyopathy

RCM is a rare form of cardiomyopathy accounting for ~5% of diagnosed cardiomyopathies. It is the least prevalent form of cardiomyopathy in children with a prevalence of one per million children [41]. Although RCM can be present at any age and is the rarest form of cardiomyopathy in children and adults, it has the poorest prognosis of any heart muscle disease in children, with a mortality of 50% within 2 years of onset and 75% within 5 years of onset of disease. 

Cardiac troponin (cTn) is a heterotrimeric structural complex found in thin cardiac muscle filaments that plays a vital role in controlling and regulating muscle contraction and relaxation via changing the concentration of intracellular calcium available to generate a contraction. It is composed of three subunits [46]:

- cTnC: a highly conserved Ca^2+^ binding subunit;
- cTnI: an actomyosin ATPase inhibitory subunit that functions to prevent actin and myosin interaction in the absence of Ca^2+^;
- cTnT: a tropomyosin binding subunit.

During systole, the sarcoplasmic release of Ca^2+^ from the L-type Ca^2+^ channels found in the T-tubules of cardiomyocytes allows the binding of Ca^2+^ to the highly conserved cTnC. This interaction promotes the interaction of myosin and actin to create muscle tension and exposes a hydrophobic cleft within the N-domain of cTnC to allow for the regulatory binding of residues 148 to 163 of cTnI. cTnI binds to cTnC and cTnT within this structural complex to form a molecular switch [46]. During systole, cTnI binds tightly to the troponin subunit and actin to inhibit the interaction between the myosin and actin filaments, ultimately leading to muscle relaxation. During systole, Ca^2+^ binds to cTnC displacing cTnI from actin and tropomyosin to allow for contraction [47]. cTnI possesses a 30-amino acid N-terminal extension that contains two protein kinase A phosphorylation sites that allow for the inhibitory binding of this subunit. The activation of these protein kinases leads to enhanced cross bridging and an increased uptake of calcium into the sarcoplasmic reticulum, allowing for muscle relaxation [41].

For the most part, the molecular bases of the pathogenesis of primary and secondary RCM differ in the genes targeted by the mutations. Both types of RCM are associated with familial inheritance, but primary RCM is more commonly associated with an autosomal dominant mode of inheritance. Several genes have been noted to be associated with primary RCM, most of which encode for proteins sarcomere, Z-disk, cytoskeleton, or intermediate filament network [48]. This disease is mainly caused by the accumulation of desmin and collagen type 3, and the genes affected with the familial type of RCM include troponin (cTn), myosin light chain (MYL), desmin (DES), myosin binding protein (MyBP), myopalladin (MYPN), and TNNI3 [41]. Mutations affecting the cardiac troponin I gene are the most commonly found sarcomeric mutations in primary RCM. These mutations can either target different domains on cTnI itself or impact TNNI3, which is vital for the production of a viable cTnI end-product. The result of the different clusters of mutations found in FRCM is a nonviable cTn complex that is incapable of inducing muscle relaxation of cardiomyocytes. This leads to a stiff ventricle and an impaired diastolic function, inducing the characteristic restrictive physiology of RCM.

### 2.3. Dilated Cardiomyopathy

Dilated cardiomyopathy (DCM) is defined as a congestive cardiac dysfunction characterized by an impaired systolic function and a dilation of either the left ventricle or both ventricles of the heart in the absence of any coronary artery disease or physiologic changes sufficient to explain the dysfunction [49]. The diagnosis of DCM is based on clinical assessments of cardiovascular function. The main criteria for a diagnosis of DCM are an ejection fraction < 45% and left ventricular dilation > 2.7 cm/m^2^. More than half of DCM cases are considered to be idiopathic, while the other half have been linked to other disorders such as ischemic, hypertensive, infectious, toxic, and autoimmune cardiomyopathies [43].

#### Molecular and Genetic Basis of Dilated Cardiomyopathy

DCM is the most prevalent form of all cardiomyopathies, one of the most common causes of heart failure, and the most common indication for heart transplant [50]. The epidemiology of this condition is complex due to the ever-evolving definitions and classification of these diseases. In a 2013 review, however, the most accurate estimation of the prevalence of DCM was determined to be more than 1 case per 250 individuals [49]. In a 2010 Global Burden of Disease study, the mortality of cardiomyopathies globally was estimated to be 5.9 per 100,000 global cases. This equates to approximately 403,000 cases globally [51].

Another study based in the United States of America concluded that the burden of disease of DCM in the US was approximately 10,000 deaths and 46,000 hospitalizations annually [52].

Idiopathic DCM (IDCM) is a multifactorial disease affected by age, gender, ethnicity, and the presence of other cardiac abnormalities, all of which could lead to an earlier onset of the disease. Familial and nonfamilial forms of IDCM have been reported, with some studies citing up to 150 genes linked to the pathogenesis of IDCM [49]. Studies have shown that the genes most commonly altered in IDCM are those encoding cardiac sarcomeric and cytoskeletal genes, most of which are inherited in an autosomal dominant pattern [53]. Alterations in the TTN gene have been documented in up to 25% of IDCM cases, making it the most common culprit in DCM pathogenesis. TTN is a gene located on chromosome 2 that encodes a 4 megadalton protein called titin. Titin forms a polypeptide that connects actin and myosin filaments in an elastic way, allowing for the proper contraction of smooth and skeletal muscles [54]. Titin has multiple regions and bands including the N-terminus, which gives the sarcomere its structural integrity by anchoring itself in the Z-disc, and the I-band region, which gives the sarcomere its elastic properties [53,54]. Most mutations in DCM, however, are frameshift or nonsense mutations targeting the A-band region of TTN. Mutations in this region affect Titin’s ability to act as a biomolecular scaffold. Such TTN variants have been linked to malignant ventricular arrhythmias, while other variants such as missense TTN variants have been deemed largely benign [49]. Finally, studies have shown that prognosis in nonsense TTN variants is negatively impacted by excessive alcohol use and repeated viral infections, hinting at a possible interplay between genetic factors and superimposed stress to induce cardiomyopathies such as DCM [53]. The second most common mutation found in DCM cases is a mutation in the LMNA gene, found in 4–6% of cases [55]. LMNA codes for lamins A and C, which are intermediate filaments that assemble heterodimers and tetradimers to stabilize the nuclear lamina as well as serve as an anchoring point for chromatin. Loss of function mutations in the LMNA gene associated with DCM cases have been confined to the rod domain, a highly conserved domain that supports the formation of higher-order polymers [55,56]. LMNA-DCM mutations are the most studied and outlined mutations in the DCM cascade despite its lower prevalence. The sheer number of studies conducted on this type of DCM mutation might be correlated with the high mortality rate of LMNA-DCM (12% at 4 years) or with the fact that it is considered to be one of the more aggressive genotypes of DCM [49]. Less prevalent genes affected in DCM include structural cytoskeletal Z-disk genes (DES, DMD, Filamin C, VCL), desmosomal genes (PKP2, DSP, DSC2), sarcomeric genes (MYH, MYBP, TNN), and ion channel-related genes (SCN5A, RYR2) [49,55]. Alterations in any of these DCM-associated genes halt the production of viable proteins that are vital for the proper contractility and elasticity of the cardiac muscle. Impairment of contractility and elasticity of cardiac muscle induce the physiologic basis of DCM by reducing cardiac output (systolic dysfunction) and reducing diastolic filling ability of the ventricles (leading to ventricular dilation), respectively [53]. 

### 2.4. Hypertrophic Cardiomyopathy

Hypertrophic cardiomyopathy (HCM) is a common genetic cardiac disease with a global distribution, spanning more than 50 countries and affecting multiple racial and ethnic origins [57]. It is the most common heritable cardiomyopathy, with a prevalence of 1 in 500 people or 0.2% of the general population. This equates to more than 600,000 individuals affected in the United States alone [58]. HCM is a myocardial disease characterized by an increase in cardiac mass, most notably the left ventricle, as a result of hypertrophic remodeling [59]. It is clinically and genetically heterogeneous, with more than 11 genes being linked to the disorder [60]. HCM is considered to be a diagnosis of exclusion given that long-standing hypertrophy and infiltrative disease can also lead to cardiac hypertrophy. Olivtto et al. broadly defined HCM as a “pathologically enhanced cardiac actin–myosin interactions, with core pathophysiological features that include hyper-contractility, diastolic abnormalities, and dynamic left ventricular outflow tract (LVOT) obstruction”, and although more than 30% of HCM cases occur without and LVOT obstruction, the prognosis is worse in HCM associated with LVOT obstruction [59,61].

#### Molecular and Genetic Basis of Hypertrophic Cardiomyopathy

Genetic studies have linked HCM to more than 11 or more genes that encode contractile proteins essential for the function of the sarcomere or the Z-disc. HCM is most commonly transmitted through autosomal-dominant inheritance, meaning that an offspring of an affected individual has a 50:50 probability of developing HCM [62]. This stresses the role of familial screening for HCM after an individual has been diagnosed with the disease to identify family members who do not currently have left ventricular hypertrophy (LVH) but are genetically prone to developing this disease [57,62]. The most commonly affected gene and the first gene to be identified in HCM is MYH7, which encodes ß-myosin heavy chain, an essential structural component of the sarcomere. Different mutations of MYH7 and multiple other genes have been linked to the disease since then, with the defining characteristic being an abundance of novel mutations in the 11 genes mentioned, specific to individuals or individual families [62,63]. Studies have shown that the prognosis of HCM seems to be independent of the specific gene or mutation affected in each HCM case [57]. Mutations in MYH7 and MYBPC3 comprise more than 70% of the variants seen in HCM cases. Multiple other mutations have been identified as causative factors of HCM, most notably TNNT2, which encodes for troponin T and accounts for <5% of the total number of pathogenic variants in HCM cases [64]. The predominant type of mutation overall is missense mutations, while MYBPC3 variants often display nonsense mutations that cause a premature stop in protein transcription [63]. Regardless of the mutation type, two main pathophysiologic mechanisms of progression of HCM have been proposed: the Haploinsufficiency model and the “poison protein” hypothesis. The predominant hypothesis of the pathogenesis of HCM is the poison protein hypothesis, which suggests that truncated mutant proteins incorporate into the sarcomere and display a dominant negative effect in the myofibrils, while the less accepted Haploinsufficiency model proposes that the defining characteristics of HCM are the product of an insufficient amount of sarcomeric protein [58,64]. Both proposed models of pathogenesis, however, lead to a reduced contractile function, secondary to altered or reduced actin-myosin interactions. This decrease in contractility causes a reduced cardiac output, which is met with an inappropriate compensatory hypertrophic remodeling and fibrosis, which leads to a higher degree of diastolic dysfunction, the hallmark characteristic of HCM [61,63].

## 3. Heart Failure

Heart failure (HF) is a progressive and complex pathological condition caused by both genetic and environmental factors [65]. It is defined as a clinical syndrome where the heart fails to pump blood at a rate commensurate with the body’s metabolic requirements [66]. The global prevalence of heart failure is estimated to be 64.34 million cases (8.52 per 1000 people), accounting for 9.91 million years lost due to disability (YLDs) [67]. In the United States, HF is estimated to affect 6.2 million American adults, with an estimated incidence of 21 per 1000 populations aged 65 years old [68]. The global cost of treating heart failure patients annually was estimated at USD 108 billion, of which 60% represented direct medical costs [69,70]. In the US, annual healthcare expenditure related to HF patients was estimated at USD 43 billion and is expected to reach USD 69.7 billion by 2030 [71]. HF is characterized by shortness of breath and fatigue at exertion or rest that is attributed to structural and functional changes in the heart and divided into two broad categories: systolic (impaired ventricular contraction) and diastolic (impaired ventricular relaxation). Systolic or diastolic HF could involve the dysfunction of the right ventricle, left ventricle or both. HF failure may result from a monogenic precursor such as in dilated cardiomyopathy (DCM), hypertrophic cardiomyopathy (HCM), arrhythmia such as long QT syndrome, or Brugada syndrome [65]. In other cases, HF can be caused by complex polygenic conditions such as coronary artery disease (CAD), diabetes mellitus (DM), or hypertension and can also result from chemotherapeutic drugs or toxic agents such as alcohol, or rarely due to pregnancy [72].

### The Molecular and Genetic Basis of Heart Failure

During the early stages of heart failure, several neurohormonal compensatory mechanisms are activated: the renin–angiotensin system, the adrenergic nervous system, cytokines, and other modulators to maintain the cardiac hemostasis, allowing the patient to remain asymptotic for a very long time. During this time, the heart undergoes structural and functional remodeling and eventually transitions from early- to late-stage heart failure [73]. Moreover, the cardiomyocyte increases in size and becomes hypertrophic. Remodeling is the heart’s process of maintaining cardiac function when subjected to physiological stress. However, when this stress persists for a very long time, the cardiac contractility declines as the heart muscles continue to increase in thickness and size, eventually resulting in decompensated and symptomatic heart failure [74]. Significant progress in this field has also been made with the help of transgenic and knockout animal studies. These studies investigated the effects of gene defects and how the suppression and/or activation of specific genes can alter neurohormonal pathways that could initiate pathological cascades and result in subsequent cardiac injury.

Over the past several years, genetic studies have laid important groundwork in identifying genetically inherited conditions that could result in heart failure. For instance, Liew et al. mapped the genes that are involved in familial cardiomyopathies to their chromosomal loci and their molecular pathways that are involved in heart failure [65]. Any of the precipitating events can activate the following pathways, eventually leading to heart failure (Table 1).

**Figure 1:**
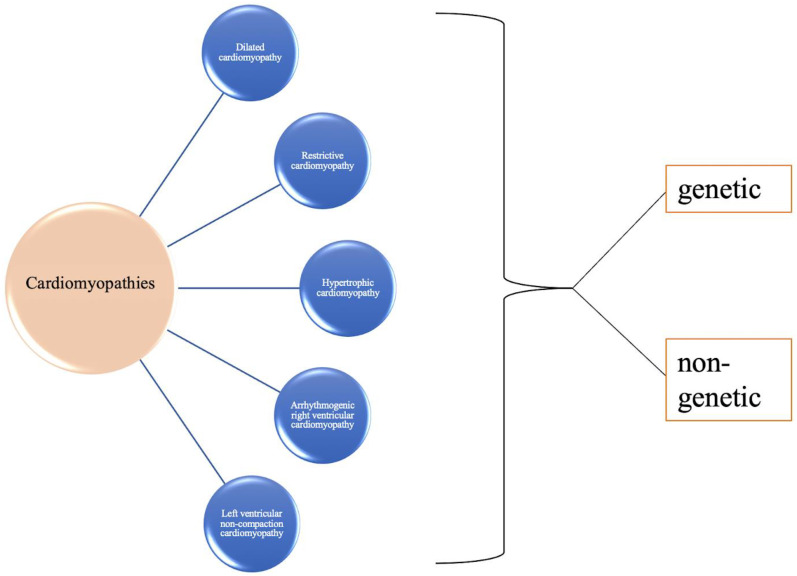
Cardiomyopathies are classified into five groups. All groups could be caused by genetic and nongenetic factors. Adopted from Czepluch et al. [76].

This groundbreaking research has revealed how genetic mutations can alter the structure and function of cardiomyocytes and how defective cardiac cell components can lead to cardiomyopathies and eventual heart failure. Cardiomyopathies are one of the leading causes of heart failure, and based on morphological and structural phenotype, they can be divided into five types: HCM, DCM, RCM, arrhythmogenic right ventricular cardiomyopathy (ARVC), and unclassified cardiomyopathy such as the left ventricular noncompaction cardiomyopathy [75]. These five entities are further categorized into familial (genetic) or nonfamilial (nongenetic etiologies); however, a clear distinction between the inherited and acquired heart failure causes remains a challenge (Figure 1).

Several mechanisms of inheritance have been identified in patients with heart failure. Autosomal-dominant inheritance is the predominant mode of transmission; however, other mechanisms such as X-linked, autosomal recessive, and mitochondrial inheritance still account for other causes of familial heart failure syndromes. Furthermore, dominant mutations can occur de novo in patients, with a 50% chance of being passed on to the offspring [77]. Heart failure vulnerability can also be affected by high-frequency but low-penetration genetic variation. In this case, variable gene expression interacts with external factors to predict HF vulnerability and thus should be viewed as a complex syndrome [78].

**Figure 2:**
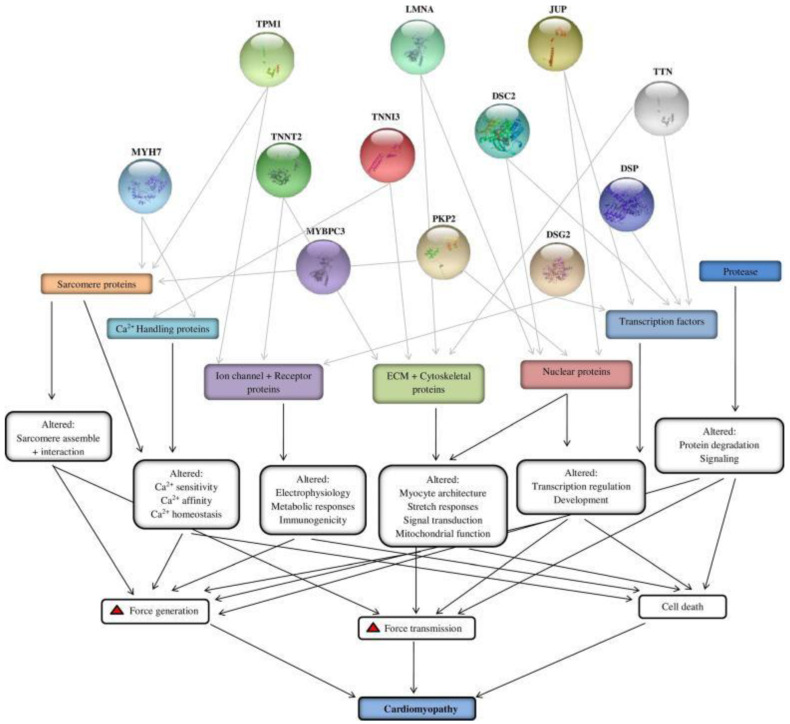
Important genes involved in cardiomyopathy and their effect on the structure and function of cardiomyocytes, adopted with permission from Kaviarasan et al. [77].

About 100 gene mutations can cause different variants of cardiomyopathies that have been discovered recently [79] (Figure 2). Most of these genes are linked to DCM, HCM, ARVC, and RCM. These genes play an important role in the pathobiology of cardiomyopathies associated with HF (Table 2). Identified genes encode important structure for the cardiomyocyte (e.g., cytoskeletal and sarcomeric genes), cardiomyocyte nucleus (e.g., laminA/C), and calcium handling (e.g., phospholamban). The most frequent mutations are mutations in lamin A/C, reported in about 7.3% of patients with DCM [80].

Each cardiomyopathy phenotype can be caused by mutations in one of the several different genes described, and even mutations in the same disease gene type can cause different disease types. For instance, although HCM, DCM, and ARVC are considered distinct cardiomyopathies, studies have found that mutations in genes that encode sarcomeric protein not only cause HCM but also can lead to DCM [81]. Similarly, according to several studies, a single mutation can cause both ARVC and DCM. These findings suggest that diseases can share genetic causes despite differing clinical phenotypes and that gene mutation alone does not adequately explain the clinical phenotype, but rather that the disease derives from an interaction between genes, neurohormonal pathways, and environmental factors, emphasizing the importance of understanding the role of network medicine and reinforcing that focusing only on the immediate genetic cause is insufficient for understanding the disease pathway. More research in this field is needed to assess the full genetic background and identify modifier mechanisms for potential clinical interventions, even in diseases that appear to be primarily explained by genetic factors [81].

## 4. Coronary Artery Disease

Coronary artery disease (CAD) is the world’s leading cause of death and is expected to remain so for the next 20 years. Globally, CAD affects approximately 126 million people (1655 per 100,000), or approximately 1.72% of the world’s population. Ischemic heart disease (IHD) alone was estimated to cause at least 9 million deaths in 2020 [82]. Men were affected more frequently than women, and the onset typically began in the fourth decade and increased with age. IHD is becoming more common worldwide. The current prevalence rate of 1655 per 100,000 population is expected to increase to 1845 by 2030. Hospitalizations, treatments, clinic visits, revascularization, and emergency visits all contribute to the financial burden of CAD [83]. In the United States, more than 300,000 people die from sudden cardiac death each year. About 80% of sudden cardiac deaths are caused by CAD, while the remaining 20% are caused by other diseases including left ventricular hypertrophy, cardiomyopathy, long QT syndrome, aortic valve disease, and Brugada syndrome [84]. According to the World Heart Federation, the global cost of CVD in 2010 was approximately USD 863 billion, with the figure expected to exceed USD 1 trillion by 2030. In countries such as the United States, the cost of IHD approaches 1–1.5% of GDP, with costs per episode of IHD exceeding USD 5000 [85]. Genetic studies for CAD/MI are lagging behind when compared with other cardiac disease genetic studies. The main reason for the field’s limited success in CAD/MI genetics is that CAD/MI is a complex disease that is thought to be caused by many genetic factors, environmental factors, and interactions between these factors. Indeed, advanced age, smoking, male gender, hypertension, diabetes mellitus, obesity, family history of CAD or MI, high fatty diet, infectious agents, elevated plasma total and low-density lipoprotein (LDL) cholesterol, elevated plasma triglycerides, and reduced plasma high-density lipoprotein (HDL) cholesterol have all been found as direct risk factors for CAD/MI [86,87].

Family history is one of the most important risk factors for CAD/MI. Twin studies indicate that genetic factors play a role in the development of CAD and MI. All of this lends support to the hypothesis that genetic factors play a role in the development of CAD and MI. A high death rate, late-onset CAD and MI, and phenocopy complications in families all pose significant challenges in the genetic dissection of this important disease [88].

We can categorize the genes linked to complex human diseases such as CAD and MI into three groups: disease-causing genes, susceptibility genes, and disease-linked genes. When disease-causing genes mutate, they are directly responsible for disease pathogenesis [89,90]). The mutations are clearly described as the primary cause of the disease in this case. LQTS is caused by mutations in the potassium channel genes KCNQ1 and KCNH2, and Brugada syndrome is caused by mutations in the cardiac sodium channel gene SCN5A. Disease-causing genes have high predictive values and can be used for genetic testing directly [84,91] (Table 1).

## 5. Pericarditis

Acute pericarditis is inflammation of the pericardium with or without pericardial effusion. It can be clinically diagnosed with two of the following criteria: a. chest pain—typically sharp and pleuritic, b. pericardial friction rub, c. electrocardiogram changes with new widespread ST elevation or PR depression, d. pericardial effusion. Acute pericarditis is the most common inflammatory heart disease in the world. Pericarditis is responsible for 0.1% of all hospital admissions and 5% of emergency room admissions for noncardiac chest pain [92]. A Finnish national registry showed a standardized incidence rate of hospitalizations for acute pericarditis of 3.32 per 100,000 person years [93]. Pericarditis can occur in infectious and noninfectious forms. The most common form of infectious pericarditis is viral pericarditis, which comprises approximately 80–85% of all pericarditis cases [94]. In the rest of the world and developing countries, tuberculosis (TB) is the most common cause of pericarditis [95].

### The Molecular and Genetic Basis of Pericarditis

Acute and chronic pericarditis are characterized by inflammation of the pericardial sac. The inflammatory response arising in pericarditis is a normal response to acute damage to the mesothelial cells of the pericardial layers [96]. This response is thought to be initiated by a virus or another trigger with the capability to stimulate the inflammatory response, then amplified through the NACHT, leucine-rich repeat, pyrin domain-containing protein 3 (NLRP3) inflammasome [97]. The NLRP3 inflammasome is a macromolecular structure located in the cytosol of primarily neutrophils and macrophages. It is made from three different components: (i) a sensor, NLRP3; (ii) an adaptor or scaffold protein, the apoptosis-associated speck-like protein containing a caspase recruitment domain (ASC), and (iii) an effector protein, caspase-1, that cleaves pro-IL-1β and pro-IL-18 to their active forms and is responsible also for gasdermin D channels for the extracellular release of IL-1β and IL-18 [98]. The activation of the NLRP3 inflammasome happens in two steps in most cells. The first step is a priming step at the time of tissue injury through the release of danger-associated molecular patterns (DAMPs), which are responsible for the transcription and translation of the components of the NLRP3 inflammasome [98]. The next step activates the inflammasome by oligomerizing the three components responsible for the typical stellate structure [99]. Interleukin-1 is an inducible cytokine that exists in two forms: IL-1α and IL-1β. IL-1β is partially constitutive and partially induced as pro-IL-1β. This process requires the inflammasome to be activated and secreted by caspase-1. The inflammasome binds the IL-1 receptor (IL-R1), which allows IL-1β to function as a true cytokine. IL-1α is present in mesenchymal cells as pro-IL-1α. Caspase-1 is not required for its cleavage. In addition, pro-IL-1α formation does not require its cleavage either. Hence, being able to be functionally active in its precursor form allows pro-IL-1 to signify the beginning of a pro-inflammatory response upon its release from the cytosol. Moreover, IL-1α has been shown to contribute significantly to early inflammatory signals after neutrophil chemotaxis, whereas IL-1β was responsible for macrophages’ recruitment [99].

There have been several studies that illustrated the contribution of genetics and immunity to pericardial diseases. For instance, pericarditis recurrences were shown to occur in almost half of pericarditis patients with known associated autoimmune diseases [100]. Cantarini et al. showed a pattern of genetic predispositions in patients with recurrent pericarditis and autoimmune conditions such as familial Mediterranean fever and tumor necrosis factor receptor-associated periodic syndrome (TRAPS). Familial Mediterranean fever tends to be associated with variants in the MEFV gene. The MEFV gene is known to be involved in interleukin-1β processing. In fact, pericarditis was noted in all 4 monogenic interleukin-1-mediated auto-inflammatory diseases including familial Mediterranean fever (FMF), cryopyrin-associated periodic syndrome (CAPS), TNF receptor-associated periodic syndrome (TRAPS), and mevalonate kinase deficiency (MKD) [101]. Claire et al. supported these findings in their study by discovering that 7.8% of patients with recurrent pericarditis demonstrated variants of MEFV and concluded that the presence of heterozygous variants of MEFV could be associated with an increased risk of the disease [101]. Tumor necrosis factor receptor-associated periodic syndrome (TRAPS) is associated with mutations in the TNFRSFIA gene. More than 40 discrete mutations were discovered in TNFRSF. TNFRSFIA is a transmembrane glycoprotein made up of an extracellular region distinguished by its distinctive pattern of four cysteine-rich domains, a transmembrane region, and an intracellular death domain, which supports signaling. Moreover, Dode et al. discovered two patients presenting with recurrent pericarditis as the single clinical manifestation of TRAPS whilst carrying the low-penetrance mutations R92Q and P46L [102].

## 6. Myocarditis

The incidence of acute myocarditis reached 1.5 million cases globally in 2013. Moreover, it was seen in association with 5% of patients with viral illness [103]. It tends to occur in females and males equally. In addition, higher rates are seen in the younger population, although it was distributed equally over numerous races [104]. Myocarditis is defined as either localized or diffuse inflammation of the myocardium and can be divided into acute, sub-acute, or chronic [105]. Approximately half of myocarditis cases tend to be idiopathic as the cause is unknown. However, in known causes, infectious and noninfectious causes contribute to the etiology of acute myocarditis. The most common form of infectious myocarditis is viral, and of note is the Coxsackie B virus, an enterovirus. Other viruses include HIV, adenovirus, hepatitis C, and COVID-19 [106]. In addition, parasites, bacteria, helminths, and fungi can also cause acute myocarditis. The noninfectious causes include sarcoidosis, systemic lupus erythematosus, polymyositis, dermatomyositis, and cocaine abuse.

Myocarditis is known to be due to interactions between the immune system and environmental factors. Studies have shown that genetic heterogeneity tends to play a role in the varied immune responses to viral infections and hence in the development of viral myocarditis. However, Belkaya et al. failed to find a relationship between TLR3 and STAT1 deficiency and viral myocarditis [107]. Patients with a severe clinical presentation of myocarditis including presentations with heart failure or ventricular arrhythmia tend to display increased levels of cytokines such as IL-1ß, IL-17, and TNF. This inadequate immune response is associated with mutations of the major histocompatibility complex genes, in particular HLA-DR4. Furthermore, genetic mutations in structural proteins are also shown to contribute to a weak myocardium and hence increase its susceptibility to inflammation and damage [108]. For instance, dystrophin mutations yield an amplified vulnerability to sarcomere rupture and increased viral titers. However, mutations in structural proteins such as SCN5A and BAG3 are associated with positive outcomes [109].

## 7. Clinical Interventions Using the Molecular and Cellular Pathways to Treat Cardiovascular Diseases

### 7.1. Antibodies

Antibodies are defined as proteins that bind to epitopes on the surface of an antigen to elicit an immune reaction. As a targeted therapy for cardiovascular disease antibodies can be divided into monoclonal antibodies (mAbs) and bispecific antibodies (bAbs) [110].

mAbs have shown great benefit in the cardiovascular field. Schwartz et al. reported that the use of alirocumab, a PCSK9 inhibitor, significantly reduced the risk of recurrent ischemic accidents and hospital stay. Patients with higher baseline LDL-C (>100 mg/dL) levels gained greater benefit than patients with lower baseline LDL-C [111]. Evidence showed that anakinra, a monoclonal antibody against Il-1 receptors, improved coronary and left ventricular function in patients with CAD [112]. Lee et al., Gundlach et al., and Lum et al. provided evidence for the effectiveness of bAbs in the treatment of ichemic myocardial cells in animal models [113,114,115]. In patients with aortic valve calcification (AVC), the monoclonal antibody denosumab was shown to have protective effects against vascular calcification in a mouse model of osteoporosis and to reduce the calcification of porcine valve interstitial cells (pVICs) [116,117]. For patients with refractory idiopathic recurrent pericarditis, anti-IL-1 monoclonal antibodies such as anakinra, canakinumab, and rilonacept have shown the highest effectiveness. Anakinra has the shortest half-life and hence yields higher safety in cases of serious infection, and it is the only drug for which randomized clinical trials illustrated supporting evidence [118].

### 7.2. Gene Therapy

**Figure 3:**
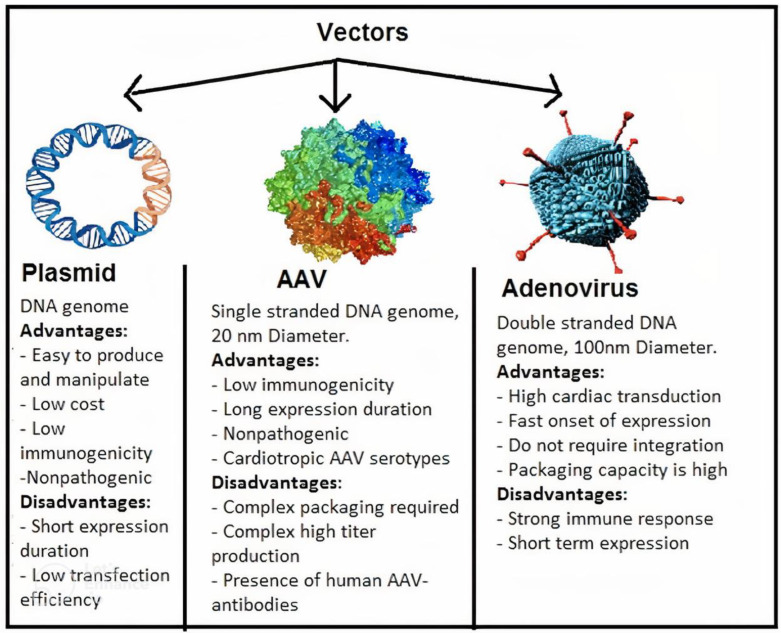
Vectors that are commonly used in cardiovascular gene transfer and their specific characteristics. Naked plasmid DNA, adeno-associated viruses (AAV), and adenovirus have been used in 18.2%, 5.2%, and 23% of the 1.902 registered gene therapy clinical trials, respectively (see www.abedia.com/wiley/vectors.ph for further information accessed on 16 August 2022).

Cardiac gene therapy is defined as a therapeutic drug delivery system that uses cardiac-targeted technology or viral vectors delivery systems for the biological administration of drugs and interventions [119]. Among heart cells, cardiomyocytes have been identified as the therapeutic target to treat systolic and diastolic heart failure, restore cardiac function, reverse remodeling, and restore electric stability and metabolism. Formulated DNA (plasmid) and RNA microRNA) are used to correct key defects in molecules that conventional drugs are unable to correct by intervening directly at the genetic and molecular levels in the diseased cardiomyocyte. The biology of the DNA/RNA product determines the classification (i.e., inotropic) using the cell’s own transcriptional and translational machinery. Since the first report of in vivo gene transfer to the myocardium, there has been a series of advances that have driven the evolution of cardiovascular disease gene therapy to the brink of clinical reality [120]. Among viral and nonviral vectors developed for system and organ delivery of gene therapy, recombinant adeno-associated virus (rAAV) is regarded today as the most efficient gene transfer agent. Because of their safety and efficiency in transducing both nondividing and dividing cell types, rAAV are used today in more than 99% of the clinical trials worldwide [119,121] (Figure 3).

Possible gene targets in the heart are calcium and beta receptor signaling as well as factors that enhance blood vessel growth (i.e., therapeutic angiogenesis). Gene therapy’s main goal is to help patients refractory to traditional therapies. There are several reported techniques for cardiovascular gene delivery, and these include myocardial injections, intracoronary perfusion, and epicardial painting, with each having its own advantages and disadvantages [122]. Some human and animal preclinical trials studying several angiogenic growth factors including VEGF, FGF, HGF, PDGF, and HIF have shown increased capillary growth, increased myocardial perfusion, and improved exercise capacity in participants with coronary artery disease (CAD) [123,124,125,126]. Nevertheless, some of these studies on CAD lack a placebo group, which signifies that further studies are still needed to confirm its efficacy and safety [127]. There are only a few studies on gene therapy for heart failure, of which some showed positive results like improved left ventricular function [128] (Table 3), whereas other trials showed no improvement in the clinical course of heart failure [129]. A gene-editing technology, CRISPR (clustered regularly interspaced short palindromic repeats), has demonstrated huge benefits in cardiovascular targeted therapy. This technique can be used to correct mutations in hypertrophic cardiomyopathy (HCM), nonischemic cardiomyopathy through interfering with the phospholamban/Ca+ homeostasis as well as mutations that cause infiltration of the myocardium [110].

### 7.3. Stem Cell Therapy 

The most commonly used stem cell therapies in trials to treat heart diseases include embryonic, tissue-specific, mesenchyme, induced pluripotent, and umbilical cord blood. These cells are used in efforts to replace damaged and generate new heart cells and improve cardiac function. Although the idea carries great potential, there is still limited evidence of the effectiveness of this therapy [148].

### 7.4. Other Therapeutic Options

In patients with AVC, Chen et al. report that zinc is a novel inhibitor of calcific aortic valve disease [149]. They illustrate that the zinc transporters ZIP13 and ZIP14 are regulators of in vitro calcification and osteogenic differentiation in human valve interstitial cells (hVICs) [149]. This study warrants further investigations to see whether zinc plays a major role in slowing the progression of AVC in vivo. Wang et al. showed that melatonin improves AVC through opposition to the osteogenic differentiation of hVICs in vivo and in vitro [150]. Melatonin reduced levels of CircRIC3 (procalcification circular RNA), which functions as a miR-204-5p (microRNA) sponge to positively control the expression of dipeptidyl peptidase-4 (DPP-4), a procalcification gene [150]. Moreover, the overexpression of CircRIC3 halted melatonin’s inhibitory effects on hVIC osteogenic differentiation [150]. Therefore, it can be inferred that AVC can be regulated via this pathway through the induction of melatonin [150].

## Figures and Tables

**Table 1:** Changes in gene expression and their functional role in the failing heart.

Gene Name and Functional Role	Gene Expression Changes in Heart Failure	Isoform Switch I the Failing Heart
**Structural**		
TNNT1 *, TNNC1 *, and TNNI1 *	↑	No change
Tropomyosin *	↑	TMP1K
MYH6	↓	MYH6 to MYH7
MYH7 *	↑	MYH6 to MYH7
MYBPC3 *	No change	No change
ACTC1 *	↑	No change
Sarcoglycan, delta *	No change	No change
Dystrophin *	Unknown	No change
Desmin *	↑	No change
Metavinculin	Unknown	No change
Muscle LIM protein	↓	No change
Actinin, alpha	↑	ACTN1 to ACTN2
Titin *	No change	N2BA to N2B
Lamin A/C	↓	No change
**Energy production**		
Succinate dehydrogenase complex *	Unknown	No change
**Calcium handling ion channels**		
Phospholamban	↑	No change
SUR2A	Unknown	No change
SCN5A	↓	No change
**Other**		
Ankyrin repeat domain 1	↑	No change
Thymopoietin	Unknown	No change
RNA-binding motif 20 *	Unknown	No change
LIM-binding domain 3 *	Unknown	No change
Tafazzin *	Unknown	No change

ACTC1, actin, alpha, cardiac muscle 1; MYBPC, myosin binding protein C; MYH, myosin heavy chain; SCN5A, sodium channel, voltage-gated, type V, alpha subunit; SUR2A, sulphonylurea receptors 2a; TNNC1, troponin C type 1; TNNI1, troponin I type 1; TNNT1, troponin T type 1. * Genes marked by an asterisk represent genes for which a causal role has been shown in large pedigrees (*n* > 3 affected) and/or in independent reports.

**Table 2:** Significant genes associated with different categories of cardiomyopathy with mode of inheritance [76,77].

Gene	Protein Name	Chromosome	Chromosome Location	Inheritance Type	Cardiomyopathy Form	Function
TNNT2	Troponin T	1	1q32.1	AD	HCM/DCM/RCM	Ca^+2^ dependent regulator of muscle contraction
MYH7	Beta myosin heavy chain	14	14q12	AD	HCM/DCM/RCM	Beta heavy chain subunit of cardiac myosin
MYBPC3	Cardiac myosin binding protein C	11	11p11.2	AD	HCM/DCM/RCM	Cardiac isoform of myosin binding protein found in the cross-bridge zone (C area) of A bands
TNN13	Troponin I	19	19q13.42	AD	HCM/DCM/RCM	Mediates striated muscle relaxation
TPM1	Alpha-tropomyosin	15	15q22.2	AD	HCM/DCM/RCM	Ca^+2^ dependent striated muscle contraction regulator
LMNA	Lamin A/C	1	1q22	AD	DCM/ARVC	Cardiac homeostasis
PKP2	Plakophilin 2	12	12p11.21	AR	ARVC	Plays a role in junctional plaques
DSC2	Desmocollin	18	18q12.1	AD	ARVC	Major components of Desmosome
DSG2	Desmoglein 2	18	18q12.1	AD	ARVC/DCM	Ca^+2^ binding transmembrane glycoprotein, component of desmosome between myocardial cells
DSP	Desmoplakin	6	6p24.3	AD/AR	ARVC/DCM	It is an essential component of functional desmosome
JUP	Plakoglobin	17	17q21.2	AD	ARVC	The common component of desmosome and intermediate junction
TTN	Titin	2	2q31.2	AD	ARVC/HCM/DCM	Essential for striated muscle assembly and function. Connects microfilaments.

**Table 3:** Possible gene therapy target for CAD and HF [123].

Gene Therapy Target for Coronary Heart Disease				
Molecular Target	Stage in Development	Findings	Model Assessed	Reference
Vascular endothelial growth factor (VEGF)	Clinical trials, phase 2/3 Continued safety and efficacy	Safe but not consistently efficacious with increasing myocardial perfusion. Success with secondary end points, i.e., increased exercise capacity and reduction in ischemic area	Human	Hedman et al., Gene Ther., 2009 [124] Stewart et al., Mol. Ther., 2009 [130]
Fibroblast growth factor (FGF)	Clinical trials, phase 2/3 Continued safety and efficacy	Safe but most trials have not increased myocardial perfusion. Some have improved exercise capacity and symptom alleviation	Human	Kukula et al., Am. Heart J., 2011 [125]
Hepatocyte growth factor (HGF)	Clinical trial, phase 1 Preclinical	Safe with negligible side effects from ADs; HGF in serum not detected after 35 days Increased capillary density and end-diastolic volume Improved cardiac perfusion and reduced apoptosis	Human Rat Pig	Yang et al., Mol. Biol. Rep., 2009 [131] Jin et al., Gene Ther., 2012 [132] Yang et al., Mol. Biol. Rep., 2010 [133]
Platelet-derived growth factor (PDGF)	Preclinical	Increased capillary growth and collateral formation from single naked DNA injection	Rabbit	Li et al., Microvasc. Res., 2010 [126]
Hypoxia-inducible factor (HIF1α)	Clinical trial, phase 1 Preclinical	Preliminary safety of ADs after 1 year Increased myocardial perfusion and improved LV function but no improvement in bioactivity end points	Human Pig	Kilian et al., Circ. J., 2010 [134] Heinl-Green et al., Eur. Heart J., 2005 [135]
Gene therapy targets for heart failure				
Molecular target	Stage in development	Findings	Model assessed	Reference
Sarcoendoplasmic Reticulum calcium-ATPase 2a (SERCA2a)	Clinical trials, phase 2	Decreased HF symptoms, increased functional status, and reversal of negative LV remodeling	Human	Jessup et al., Circulation, 2011 [136]
Stromal-derived factor-1 (SDF-1)	Clinical trials, phase 1/2	Safe and improved 6-min walk test, quality of life, and NYHA class	Human	Penn et al., Circ. Res., 2013 [137]
Adenylyl cyclase 6 (ADCY6)	Preclinical	Increased LV function, increased cAMP levels, reversal of dysfunctional β-AR signaling, and increased survival Improved LV contractility	Mice Pig	Rebolledo et al., Hum. Gene Ther., 2006 [138] Roth et al., Circulation, 1999, 2002 [139,140] Takahashi et al., Circulation, 2006 [141] Lai et al., Circulation, 2000 [142]
βARKct-carboxy terminal peptide from GRK2	Preclinical	Heart failure rescue Improved β-AR signaling and contractile dysfunction	Rabbit Human Cardiomyocytes	Shah et al., Circulation, 2001 [143] Williams et al., Circulation, 2004 [144]
S100A1	Preclinical	Increased reuptake SR Ca^2+^, lowered Ca^2+^ leak, enhanced cardiac function, and reversed LV remodeling	Rat Cardiomyocytes	Most et al., J. Clin. Invest., 2004 [145] Pleger et al., Circulation, 2007 [146]
Parvalbumin (PVALB)	Preclinical	Increased rate of Ca^2+^ removal and improved relaxation rate	Rat	Szatkowski et al., J. Clin. Invest., 2001 [147]

## Data Availability

Not applicable.
